# The Ketogenic Effect of Medium-Chain Triacylglycerides

**DOI:** 10.3389/fnut.2021.747284

**Published:** 2021-11-18

**Authors:** Ting-Yu Lin, Hung-Wen Liu, Tsung-Min Hung

**Affiliations:** Department of Physical Education and Sport Sciences, National Taiwan Normal University, Taipei City, Taiwan

**Keywords:** aging - old age - seniors, cognition, octanoic acid (caprylic acid) (PubChem CID: 379), decanoic acid (PubChem CID 2969), Tricaprin – Captex® 1000 (PubChem CID: 69310), Tricaprylin – Captex® 8000 (PubChem CID: 10850), ketone bodies, beta-hydroxybutyrate

## Abstract

Medium-chain triacylglycerides (MCTs) are dietary supplements that can induce ketosis without the need for a traditional ketogenic diet or prolonged fasting. They have the potential to marginally delay the progression of neurodegenerative diseases, such as Alzheimer's disease. However, there have been inconsistencies in reports of the MCT dose–response relationship, which may be due to differences in MCT composition, participant characteristics, and other factors that can influence ketone generation. To resolve these discrepancies, we reviewed studies that investigated the ketogenic effect of MCTs in healthy adults. Aside from the treatment dose, other factors that can influence the ketogenic response, such as accompanying meals, fasting duration, and caffeine intake, were assessed. Based on the available literature, four practical recommendations are made to optimize the ketogenic effect of MCTs and reduce unwanted side effects (primarily gastrointestinal discomfort and diarrhea). First, the starting dose should be either 5 g of octanoic acid [caprylic acid (C8); a component of MCTs] or 5 g of a combination of C8 and decanoic or capric acid (C10; another component of MCTs), and the dose should be progressively increased to 15–20 g of C8. Second, MCTs should be consumed after an overnight fast, without an accompanying meal if tolerable, or with a low-carbohydrate meal. Third, the addition of caffeine may slightly increase the ketogenic response. Fourth, emulsifying the MCTs might increase their ketogenic effect and alleviate side effects.

## Introduction

Aging and dementia are two crucial issues that affect people worldwide. The World Health Organization reported that 1 billion people are now aged ≥ 60 years, with 50 million having dementia. Of those with dementia, 60–70% have Alzheimer's disease (1, 2). Therefore, the development of a cost-effective intervention to mitigate brain deterioration and prevent Alzheimer's disease is crucial.

One symptom and possible cause of brain function decline later in life is glucose hypometabolism. Older adults show a 7% reduction in glucose metabolic rate in the gray matter of the brain and a 10–14% deficit in the frontal cortex compared with younger adults [(3) (R2)]. Older adults with mild Alzheimer's disease show an additional 13% decrease in global cerebral glucose metabolic rate in the gray matter compared with their healthy counterparts [(4) (R2)]. One way to compensate for a low glucose metabolic rate is to use an alternative energy source such as ketones [β-hydroxybutyrate (βHB) and acetoacetate], which have been reported to be metabolized normally in patients with Alzheimer's disease [(4) (T3)].

Medium-chain triacylglycerides (MCTs) are commercially available, inexpensive dietary supplements that can induce ketosis. Aside from serving as an alternative energy source, βHB has been reported to have health benefits similar to those observed upon calorie restriction, such as antiaging effects *via* epigenetic regulation [(5) (Pg306, 310–311)]. Therefore, an understanding of the relationship between the amount of MCTs consumed and the level of ketones in the blood is necessary to inform practical applications of MCTs. An earlier review reported an almost linear relationship between the amount of MCTs consumed, up to doses as high as 70 g, and their ketogenic effects [(6) (F6)]. However, another recent study showed a non-linear relationship between these two parameters at relatively low doses of MCTs (10–20 g) [(7) (F1A)]. This inconsistency may arise from differences in the compositions of MCTs and participants' health conditions, limitations of the study designs, and other cofounding factors (discussed in the following section). Thus, we aim to provide a brief review of the available literature on the ketogenic effect of oral MCT administration in healthy participants and related interacting factors, such as accompanying meals, fasting duration, and caffeine intake. We also provide a practical summary on how to maximize the ketogenic benefits of MCTs.

## Ketogenic Effect of MCTs

### Effect of Carbon Chain Length on the Ketogenic Effect of MCTs

The major ketogenic component of MCTs is caprylic acid (C8), followed by capric acid (C10) or lauric acid (C12) [(8) (R, F1, F3)]. Caproic acid (C6) is excluded because it is not typically consumed as a dietary supplement, partly due to its unpleasant odor. The ketogenic effect (total ketone concentration) of C8 is three and six times higher than the effects of C10 and C12, respectively [(9) (D1, F3)]. Vandenberghe et al. showed that the total plasma ketone concentration increased after the consumption of C8 for ~4 h, but not after the consumption of C10 [(8) (R5, F3)]. Other studies have shown that consuming 18–20 mL (~16–18 g) of C8 without an accompanying meal increases the plasma βHB concentration from <0.1 mmol/L to 0.5–0.6 mmol/L at 2 h after administration [(9) (F1)].

Before the results from recent studies, such as that conducted by Vandenberghe et al. [(8) (R)], came to light, all of the MCTs (i.e., C6, C8, C10, and C12) were considered to be ketogenic [(10) (I1)], partly because these molecules are rapidly absorbed and transported to the liver via the portal vein mainly as free fatty acids bound to serum albumin. In contrast, longer-chain triacylglycerides are dependent on acyl-CoA synthetase activity and the lymphatic system for absorption and are transported by chylomicrons [(11) (Pg951)]. However, only fatty acids with carbon chain lengths ≤ 8 can cross the inner membrane of the mitochondria independent of carnitine palmitoyl transferase I [(12) (T1)]. This may be why C8 has a stronger ketogenic effect than C10 and C12.

In their review, Cunnane et al. suggest the presence of a linear relationship between oral doses of MCTs up to 70 g and the maximal observed plasma βHB concentration [(6) (F6)]. However, some issues were noted after reviewing the studies cited in their figure. First, the cited studies included not only healthy adults (13, 14) but also memory-impaired adults (15) and patients with type 1 diabetes (16) and Alzheimer's disease (17). Second, the doses presented in their figure were the sum of all MCT doses (i.e., C6, C8, C10, and C12) and not just the dose of C8, which has been reported to contribute to the majority (9), if not all [(8) (R, F1, F3)], of the ketogenic response. Finally, in their analysis, Cunnane et al. did not consider whether the MCTs had been consumed with or without a meal or the number of hours the participants had fasted.

Based on the above data and other recent findings, the non-linear relationship between MCT intake and plasma ketone concentration might begin at a relatively low dose of MCTs (6 g of C8 + 4 g of C10 vs. 12 g of C8 + 8 g of C10) [(7) (F1A)]. Based on the results of several studies (7, 8, 18), it is clear that 20 g of C8 produces a significantly stronger (but perhaps not twice as high) [(7) (D2)] ketogenic response than 10 g of C8. Further studies are required to determine whether doses higher than 20 g can produce a significantly larger ketogenic response and/or a greater risk of unwanted side effects. Norgren et al. also suggested [(19) (D5)] that the dose of C8 should be limited to 15–20 g per intake to minimize potential side effects.

### Repeated Use of MCTs May Influence the Acute Ketogenic Response

It is unclear whether the repeated use of MCTs can augment the acute ketogenic response. In a previous study, Freund and Weinsier showed that repeated administration of MCTs to the same participants showed reproducible responses within narrow limits [(20) (A)]. However, a 1-month interventional study showed that the daily mean plasma βHB concentration increased from ~0.1 mmol/L to ~0.2 mmol/L after consuming ~6 g of C8 twice a day for 30 days [(21) (F1A-B)].

### Effect of an Accompanying Meal on the Ketogenic Effect of MCTs

Consuming MCTs without an accompanying meal produces a stronger ketogenic effect than with an accompanying meal that has a substantial carbohydrate content. For example, adding 50 g of glucose to 20 g of C8 (0.27 mmol/L vs. 0.10 mmol/L from control) decreased the ketogenic effect (measured as the venous whole blood βHB concentration) by 63% [(19) (T3)]. Another study showed that the plasma ketone response was ~2-fold higher after consuming C8 without an accompanying meal than with a meal [470 calories in the meal; 19.5 g of fat (36%), 24.2 g of protein (20%), and 55 g of carbohydrate (44%)] [(9) (A)]. As the amount of carbohydrate consumed with the MCTs increases, the ketogenic response decreases [(20) (R4, F4)]. Therefore, consuming C8 without an accompanying meal can maximize the ketogenic effect, i.e., it can be consumed as a replacement for breakfast or as a stand-alone snack [(9) (D2)].

Consuming carbohydrates after consuming MCTs also decreases their ketogenic effect. For instance, the subsequent consumption of sucrose suppresses the ketogenic effect of MCTs. As the amount of sucrose consumed increases, the maximal acetone concentration in alveolar air decreases [(20) (R5, D5, F5–6)].

Although it may not inhibit the ketogenic effect, consuming a low-carbohydrate meal with C8 may prolong the time required to attain the maximal plasma βHB concentration. A low-carbohydrate ketogenic breakfast with ~110 g of fat (~28 g of C8 and ~43 g of C10), 25 g of protein, and 3 g of carbohydrate can elevate the plasma βHB concentration to approximately 0.7 mmol/L at 1 h and 2 h after administration, with a peak at 6 h after administration (~1 mmol/L) [(13) (R1, F1)].

### The Effect of Fasting on the Ketogenic Effect of MCTs

after an overnight fast (~12 h), the plasma ketone concentration reaches ~0.07–0.15 mmol/L [(8, 13, 21) (T1), 13(T1), 21(T2)]. Longer fasting periods (12, 18, and 24 h) result in greater ketogenic responses (measured as acetone concentration in alveolar air) after consuming a single dose of MCTs [30 mL, 74.7% (~20 g) C8]; the measured acetone in alveolar air after 4 h was ~1, 1.5, and 2.5 μg/100 mL after fasting for 12, 16, and 24 h, respectively [(20) (R7, F7)].

### The Effect of Coffee/Caffeine on the Ketogenic Effect of MCTs

Caffeine, when taken with a high-carbohydrate breakfast and without MCT consumption, can increase the plasma βHB concentration [(22) (F2)]. Consuming 5 mg/kg of caffeine along with a high-carbohydrate breakfast (~482 kcal; 71% carbohydrate, 18% fat, and 12% protein) results in a significantly higher plasma βHB concentration after 3, 3.5, and 4 h (~150–200 vs. 50–100 μmol/L), compared with eating breakfast alone [(22) (F2B)].

Although McAllister et al. claimed in their article title that acute coffee ingestion increases the blood ketone concentration, it is not clear whether this was caused by coffee or fasting, as there was no non-coffee control condition in their study [(23) (Title, F1B)]. Combining caffeine with MCTs may indeed potentiate the ketogenic effect of MCTs, but this requires further investigation [(22) (D3)].

### Emulsification Might Influence MCT Absorption

The emulsification of MCTs with beverages increases their ketogenic effect compared with the same dose of non-emulsified MCTs (increase in the 4-h area under the curve from 0.147 ± 0.094 to 0.560 ± 0.095 mmol^*^h/L with 12 g of C8 and from 0.311 ± 0.097 to 1.320 ± 0.336 mmol^*^h/L with 18 g of C8) [(18) (F1)]. However, the effect of emulsification and the optimal technique of emulsification (e.g., using a blender) require further investigation.

### The Effect of Exercise on the Ketogenic Effect of MCTs

Although Vandenberghe et al. claimed that aerobic exercise increases the ketogenic effect of MCTs [(24) (Title)], all of the participants in their study underwent the same intervention sequence (control → MCTs → aerobic exercise → MCTs + aerobic exercise) [(24) (M, F1)]. As the study design did not include randomization or even a counterbalance, it remains unclear whether aerobic exercise indeed augments the ketogenic effect of MCTs. Additional studies with a more robust design are required to address this question.

### The Effect of Consuming Other Fatty Acids on the Ketogenic Effect of MCTs

Adding 30 g of coconut oil (<10% C8, 5–6% C10, and 41–42% C12) (25) to 20 g of C8 does not significantly increase the venous whole blood βHB concentration over a period of 6 h compared with 20 g of C8 alone [(19) (F1, T3)]. Moreover, 30 g of coconut oil alone does not significantly increase the venous whole blood βHB concentration [(19) (T3)] after 6 h compared with the control condition (30 g of sunflower oil).

### Beginning With a Low Dose and Emulsification May Reduce Side Effects

MCTs are safe at doses up to 1 g/kg [(26) (A)]. However, common side effects such as gastrointestinal discomfort [(27) (T1)] and diarrhea [(20) (R1)] do occur. If an individual is not accustomed to MCTs, then there is a significant possibility that they will experience side effects. For instance, three out of seven participants in Freund and Weinsier's study experienced abdominal discomfort and diarrhea after consuming 25 mL (~23 g) of MCTs [(20) (R1)]. To counter this, Courchesne-Loyer et al. recommend starting with 5 g of MCTs in the morning [(18) (D4)]. Emulsification may also reduce the side effects of MCTs [(18) (T2)], but adding glucose to coconut oil or C8 does not reduce adverse effects such as nausea and upset stomach [(19) (T5)].

## Practical Summary

1. Start with a low dose (5 g or 6 mL) of C8 or C8 + C10. If there are no adverse effects such as diarrhea or other abdominal issues, then increase the dose up to 15–20 g of C8 (17–22 mL). For example, if consuming an MCT product with 50% C8, increase the dose from 6 to 33–44 mL (15–20 g of C8).
2. To optimize the ketogenic effect of MCTs, consume C8 after an overnight fast, without an accompanying meal if tolerable or with a low-carbohydrate meal. After consuming C8, fast for several hours to maximize the time under mild ketosis. For example, if dinner is completed at 8 p.m., then consume C8 at 8 a.m. the next day as a breakfast substitute and wait until lunch to break the carbohydrate/protein fast.
3. Consuming MCTs with caffeine may slightly increase their ketogenic effect.
4. Emulsifying MCTs (perhaps using a blender) might increase their absorption rate and decrease the risk of adverse effects.

## Discussion

By reviewing and citing the available literature in detail, this brief article clearly identifies the discrepancies in previous studies and provides several practical recommendations on how to consume MCTs to maximize their ketogenic benefits. However, in addition to the effects of their metabolites (i.e., ketones), C8 and C10 have been shown to directly improve neural function. For instance, C8 regulates the energy balance by affecting the activity of pro-opiomelanocortin neurons [(28) (Sec3.4)]; C10 enhances mitochondrial function in neurons by activating the SIRT1 enzyme [(29) (Sec3.3.2)] and regulates astrocyte function by inhibiting mTOR [(30) (R12, F7)]; and both C8 and C10 increase neuronal GABA synthesis by increasing the glutamine supply [(31) (R5, F5)]. Thus, the optimal dose and composition of MCTs may differ from previous recommendations and from those provided hereafter in the light of other relevant factors.

As participants with diseases such as diabetes and Alzheimer's disease may have different metabolic responses, we only included studies on healthy participants. Healthy adults and elderly individuals do not seem to show major differences in their ketogenic responses to MCTs [(13) (R1, F1)]. However, further investigations of the ketogenic effect of MCTs in different populations are necessary.

## Author's Note

In-text citation: A: abstract; I: introduction; M: method; R: results; D: discussion; number after abbreviation: paragraph; T: table; F: figure; Sec: section; Pg: page.

## Author Contributions

T-YL conceived and conducted the research and collected and analyzed the data. He is the guarantor of the study and responsible for writing the manuscript. H-WL provided advice to improve the clarity of the manuscript. T-MH is the supervisor and is responsible for reviewing the manuscript. All authors have contributed to drafting the manuscript and have approved of the final version.

## Funding

T-YL was supported by the Scholarship Program for Elite Doctoral Students [Ministry of Science and Technology (Taiwan)].

## Conflict of Interest

The authors declare that the research was conducted in the absence of any commercial or financial relationships that could be construed as a potential conflict of interest.

## Publisher's Note

All claims expressed in this article are solely those of the authors and do not necessarily represent those of their affiliated organizations, or those of the publisher, the editors and the reviewers. Any product that may be evaluated in this article, or claim that may be made by its manufacturer, is not guaranteed or endorsed by the publisher.
